# Molecular Feasibility of Gene Sequencing on Lymph Node Fine-Needle Cytology Samples of Non-Hodgkin Lymphoma

**DOI:** 10.3390/ijms27135962

**Published:** 2026-07-02

**Authors:** Angela D’Ardia, Elisabetta Maffei, Sara Gaeta, Teresa Infante, Valentina Giudice, Francesco Sabbatino, Anna Di Filippo, Marco Picardi, Pio Zeppa, Alessandro Caputo

**Affiliations:** 1 Pathology Department, University Hospital “San Giovanni di Dio e Ruggi d’Aragona”, 84131 Salerno, Italy; maffeielisabetta@gmail.com (E.M.); sara.gaeta@sangiovannieruggi.it (S.G.); teresainfante84@gmail.com (T.I.); annaotantasei@gmail.com (A.D.F.); pzeppa@unisa.it (P.Z.); alessandro.caputo94@gmail.com (A.C.); 2 Haematology Department, University Hospital “San Giovanni di Dio e Ruggi d’Aragona”, 84131 Salerno, Italy; vgiudice@unisa.it; 3 Oncology Department, University Hospital “San Giovanni di Dio e Ruggi d’Aragona”, 84131 Salerno, Italy; fsabbatino@unisa.it; 4 Department of Clinical Medicine and Surgery, Hematology Section, University of Naples “Federico II”, 80131 Naples, Italy; marco.picardi@unina.it

**Keywords:** lymph node, non-Hodgkin lymphoma, next generation sequencing, molecular assessment, fine-needle aspiration cytology

## Abstract

Non-Hodgkin lymphomas (NHL) are lymphoproliferative neoplasms with a heterogenous genetic landscape that require multiple assays to be characterized. Cytological samples are frequently utilized in the diagnosis of NHL. An RNA-based assay was utilized to detect translocations and identify mutations in fine-needle aspiration cytology (FNAC) samples of NHL. Fragmentation index and RNA concentration were evaluated in 54 FNAC and eight corresponding histological samples of NHL to establish first the material adequacy before sequencing. To sequence FusionPlex Lymphoma (ArcherDX, Boulder, USA), an anchored multiplex polymerase chain reaction-based RNA targeting 125 genes was used. The sequencing data processing was entirely carried out on the ArcherDX online platform. Mutations were detected in 21 of 62 samples (34%), and in 41 of 62 samples (66%), no genomic alterations were found. The FusionPlex assay detected five BCL6 translocations (three IGH-BCL6 and two EIF4A2-BCL6), six IGH-BCL2 translocations, two IGH-CCND1 translocations, two TP53 point mutations, two MYD88 mutation and four uncommon translocations (two EIF4E3-FOXP1, one TBL1XR1-TP63, one LOC105370537-FUT8). In eight out of eight cases, there was NGS (Next Generation Sequencing) results concordance between corresponding cytological and histological samples (100% concordance). FNAC samples of NHL are suitable for molecular assessment by NGS and FusionPlex Lymphoma assay is an effective method for this purpose. NGS allows the detection of mutations and the identification of translocations on cytological samples also with scanty diagnostic material.

## 1. Introduction

Genetic features are an integral part of the contemporary classification of non-Hodgkin lymphoma (NHL). Recurrent chromosomal alterations, generally assessed through cytogenetic analysis, are crucial in defining NHL and, in many cases, represent the cornerstone for diagnosis in addition to morphology and immunophenotypic analysis. The heterogeneity of NHL is closely related to chromosomal changes: namely, numerical and/or structural abnormalities. The association of these abnormalities with specific histological patterns is significant and generally correlated with well-defined patterns and the histological architecture of the corresponding NHL. Fluorescence in situ hybridization (FISH) is the most commonly used technique to detect these specific chromosomal aberrations [1].

Beyond specific chromosomal aberrations, the introduction of HTS (high throughput sequencing-based technologies) has greatly expanded the genomic landscape of many NHLs [2]; however, although specific mutations have been identified only in some entities, such as the *MYD88* p.L265P mutation in lymphoplasmacytic lymphoma (LPL) and the *BRAF* p.V600E mutation in hairy cell leukemia (HCL). Conversely, in most lymphoid neoplasms, a more diversified pattern is observed with only a small number of variably frequent aberrations followed by a long series of unusually mutated genes. This genetic variability has prevented the development of a dynamic molecular diagnostic algorithm and alteration-target therapy as in other parenchymas [3]. However, diagnostic and prognostic aberrations with predictive impact have been identified in various entities. In the near future, with the advancement of clinical studies, NHL samples will need to be routinely sequenced to ensure patient stratification into different treatment arms specific to mutational status.

In the last few years, small biopsies or cytological samples, mainly represented by FNAC, are routinely submitted to labs of pathology with wide and articulated requests that raise the need for the accurate management and capitalization of diagnostic material for different purposes, including molecular testing. Lymphoid tissues from lymph nodes or extranodal lymphoid tissues are also submitted in the form of FNAC or core needle biopsies (CNB). Not only that, long survival patients with lymphoproliferative processes may require the reevaluation of relapses or oncoming processes on cytological or small histological samples because surgical biopsies may exceed the patients’ compliance. The aim of this study is to assess whether lymph node FNAC samples of NHLs are suitable for NGS molecular profiling using an RNA-based assay.

## 2. Results

**Figure 1:**
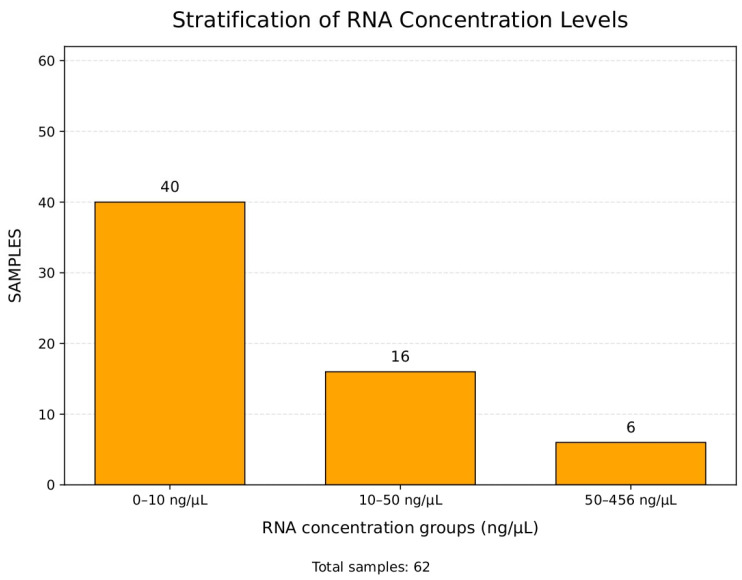
Stratification of RNA concentration levels. RNA concentrations (ng/μL) were categorized into three groups to facilitate comparative analyses: Group 1 (low concentration, 0–10 ng/μL, n = 40), Group 2 (medium concentration, >10–50 ng/μL, n = 16), and Group 3 (high concentration, >50–456 ng/μL, n = 6).

RNA concentrations (ng/μL) were stratified into three distinct groups to facilitate comparative analyses. Group 1 (low concentration) included samples with RNA levels ranging from 0 to 10 ng/μL, comprising 40 samples. Group 2 (medium concentration) encompassed RNA concentrations greater than 10 up to 50 ng/μL, with a total of 16 samples. Group 3 (high concentration) consisted of samples with RNA concentrations exceeding 50 up to 456 ng/μL, represented by six samples. These data are represented in Figure 1 Out of the 62 NHL samples analyzed, approximately 67% (42 samples) exhibited high fragmentation, which may reflect compromised RNA quality. About 21% (13 samples) showed moderate fragmentation levels, while roughly 11.3% (7 samples) possessed low fragmentation, indicating optimal RNA integrity for molecular analyses. This stratification underscores the variability in RNA quality within the cohort, emphasizing the importance of fragmentation assessment prior to downstream applications such as sequencing or gene expression profiling. Ensuring high-quality RNA is crucial for obtaining reliable and reproducible results in molecular pathology studies. The authors decided to sequence all the previously selected cases to define the robustness of the method. The sequencing metrics for each sample were satisfactory and met the predefined parameters. All samples passed the QC with a range from 86.5 to 167. Quality control metrics indicated that, across the 62 analyzed samples, the total number of fragments passing quality filtering ranged from 1,600,446 to 2,895,144. The number of successfully mapped fragments ranged from 1,596,248 to 2,807,395, and the mapping rate was greater than 98% for all samples. Mutations were detected in 21 of 62 samples (34%), and in 41 of 62 samples (66%), no genomic alterations were found among the 125 studied genes. The FusionPlex assay detected 4 point mutations and 17 translocations as follows: two *TP53*, two MYD88, five *BCL6* translocations (three *IGH-BCL6* and two *EIF4A2-BCL6*), six *IGH-BCL2* translocations, two *IGH-CCND1* translocations and four uncommon translocations (two *EIF4E3-FOXP1*, one *TBL1XR1-TP63*, one *LOC105370537-FUT8*). As noted above, eight histological cases were included in the study cohort to assess concordance with the corresponding cytological specimens. NGS results showed 100% concordance between the sequencing of histology and cytologic samples. Namely, four histological samples confirmed the presence of a genetic alteration (two *IGH–BCL2* translocations, one *TP53* mutation, and one *MYD88* mutation), whereas the other four samples confirmed the absence of pathogenic variants across the 125 genes analyzed.

**Figure 2:**
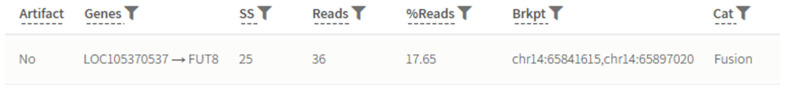
Fusion identified by ArcherDX analysis. A fusion involving *LOC105370537* and *FUT8* was detected, and the corresponding breakpoints were located on chromosome 14 (chr14:65,841,615 and chr14:65,897,020). The uncommon fusion event identified in this sample was supported by robust sequencing metrics. As shown, the SS number exceeded the established threshold (25 vs. ≥5), the number of supporting reads was 36 (threshold ≥ 10), and the variant allele frequency (VAF), calculated as the percentage of supporting reads, was 17.65% (threshold ≥ 10%).

Results are described and shown in Table 1. The identification of the fusion event with specific sample sequencing metrics by the bioinformatic pipeline is shown in Figure 2.

## 3. Discussion

Chromosomal alterations, generally evaluated by fluorescence in situ hybridization (FISH), are the traditional genetic pillars of the study of NHL. In fact, specific translocations and deletions, together with morphological and immunophenotypic analyses, represent the basis of NHL diagnosis and classification. Other than chromosomal alterations, clonality may be useful to assess malignancy through IG and TR loci rearrangements using PCR-based analyses [13,14,15]; more recently, high-throughput sequencing (HTS) has also been used [16]. However, clonality is not always indicative of NHL because dominant clones have been observed in reactive conditions, mainly associated with autoimmune processes or T-cell immunodeficiencies. Gene expression profiling (GEP) and DNA methylation analysis have also been used to identify NHL subgroups—mainly diffuse, large B-cell lymphoma (DLBCL) and “cell-of-origin” signatures [17]. Information obtained by GEP may have a role in the prognostic evaluation of NHL but seems less effective in subtype classification or in the predictive evaluation of NHL [18]. Fluorescence in-situ hybridization is the most utilized procedure to identify chromosomal aberrations, mainly involving the IG loci and different gene partners using either fusion or break-apart probes [19]. Whereas conventional FISH is effective in the identification of specific NHL entities, its main limitation is being a single test utilized on specific morphological and phenotypic indications. Despite the stabilized and diffuse cytogenetic classification of NHL, molecular assays of NHL are not routinely applied. In fact, whereas HTS technologies have detected numerous different genetic mutations in NHL [20], many of these mutations are not specific and do not have diagnostic and predictive value so far [21]. Specific mutations have been detected only in a few NHL entities, such as the *MYD88* p.L265P mutation in lymphoplasmacytic lymphoma (LPL) and the *BRAF* p.V600E mutation in hairy cell leukemia (HCL) being many of the other entities characterized by different and apparently heterogeneous and unspecific mutations [5,22]. Nonetheless, the availability of increasingly wider and more specific gene panels and a diffuse application of sequencing of NHL might detect other specific diagnostic and predictive genetic variants. In this perspective, it is conceivable that the future for NHL classification and corresponding tailored therapies for specific entities and single patients will depend on this molecular profiling [23]. The review by Martínez-Martín et al. [24] highlights the ongoing transition from conventional diagnostic approaches based on morphology, immunophenotyping, and FISH toward molecular classifications incorporating mutational profiles, copy number alterations, and structural variants. While FISH remains the reference method for detecting MYC, BCL2, and BCL6 rearrangements, the authors emphasize the limitations of immunohistochemical assessment and discuss transcriptomic signatures such as DHITsig, which can identify biologically high-risk lymphomas beyond cytogenetically defined double-hit cases. These observations support the growing role of RNA-based NGS technologies, whose high analytical sensitivity and multiplexing capabilities enable a more comprehensive molecular characterization and may improve risk stratification in aggressive B-cell lymphomas.

Lymph node histological evaluation is the gold standard for NHL diagnosis; however, in recent years, FNAC and core-needle biopsies (CNB) have been more and more utilized for the lymph node diagnosis of different processes, including NHL. These technologies are safe, rapid, cheap and flexible enough to be applied in different clinical contexts [16]. At the same time, molecular testing is routinely used on cytological samples for diagnostic and predictive purposes in different neoplasms [25]. The aim of this study has been therefore to assess the feasibility of an RNA-based assay to detect translocations and identify mutations in cytological samples of NHL. Perrone et al. [26] offer significant perspectives on host genetic factors impacting therapeutic success in DLBCL. By confirming germline variants linked to R-CHOP resistance, these investigators broadened the comprehension of elements affecting treatment efficacy beyond traditional tumor-specific biomarkers. Such data emphasize the necessity of combining host pharmacogenetic data with molecular tumor assessment, fostering the move toward sophisticated multi-omic patient stratification. Within this framework, the heightened sensitivity of contemporary *NGS* platforms, coupled with advancements in library construction and bioinformatic workflows, has improved the identification of pathogenic variants even in specimens with scanty or degraded nucleic acids. RNA-based *NGS* methodologies now permit detailed transcriptomic analysis, including fusion transcript identification, thus increasing the array of predictive biomarkers for clinical management. Nonetheless, the observations by Perrone et al. suggest that achieving the full promise of precision medicine in DLBCL entails merging transcriptomic results with genomic and pharmacogenetic context to evaluate both intrinsic tumor characteristics and host-related variables that determine clinical outcome.

As reported above, for this retrospective research study, 62 NHL samples were utilized, consisting of 54 cytological and eight corresponding histological samples. Despite a subset of samples exhibiting a high degree of fragmentation, as reported in Table 1, all samples were successfully sequenced. The absence of inadequate samples confirmed the robustness of the methodology and the validity of the cytological and histological specimens. As previously reported [19,20], RNA extraction from FFPE of solid tumors, including NHL, is reliable, and our results are consistent with these data. Conversely, the utilization of FNAC samples is effective in solid tumors but has not been described on NHL so far [25]. Therefore, the present data support the feasibility of gene sequencing on LN-FNAC of NHL, whereas there are no other studies to compare the present experience. From a technical point of view, the protocol used was relatively easy to apply, and the analysis on the online platform was very intuitive. The same positive experience was described from another group [25], who also noted that this kit is efficient when dealing with limited samples.

As previously reported, in the present series, 21 cases were mutated (34%) and 41 were not mutated (66%). Crotty et al. [25] reported mutations in 24 out of 41 tested NHL (58%) versus 17 wild types (42%). The percentages obtained are relatively different, with a higher mutation frequency in Crotty et al.’s cases compared to our results. These different results are justified considering that Crotty et al.’s [25] series consisted of DLBCLs, which carry a higher mutation frequency, particularly related to *BCL2* and *BCL6* rearrangements [23,24]. It is also true that, in both studies, the sample sizes were small, and a larger sample would be needed to estimate the mutation percentage in a heterogeneous NHL sample.

In two of the four uncommon translocations identified, *EIF4E3* was the fusion partner with *FOXP1*. Namely, considering that *EIF4E3* is known as a prognostic marker in ovarian and lung carcinoma and is also involved in the pathogenesis of multiple myeloma, *FOXP1* is a gene commonly involved in the lymphomagenesis of NHL. B-cells in NHL exhibit aberrant expressions of the transcription factor FOXP1, which further contributes to the clinical behavior of lymphoma and treatment resistance. The term “uncommon” here does not imply “rare” but rather that the genes involved are not among the main targets routinely tested in diagnostics. Furthermore, *FUT8*, *FOXP1*, and *EIF4E3*, when mutated or rearranged, are involved in lymphomagenesis but are not as common as *IGH*, *BCL2*, *BCL6*, *MYC*, and *CCND1* [27,28]. *LOC105370537*, on the other hand, is an uncharacterized polymorphism, meaning its involvement in cellular biological processes has not yet been described. This is why the first translocation mentioned, *LOC105370537-FUT8*, is considered uncommon. In two cases of BL, a somatic mutation of *TP53* was identified. *TP53* has a prognostic value in solid tumors and NHL, and it is often associated with aggressive behavior in corresponding neoplasms. The literature indicates that *TP53* mutations are common in B-cell NHLs, particularly present in about 33% of BL versus 100% obtained in the BLs of the present series [29]. This different percentage is justified from the limited number of BLs considered here (2 cases). The obtained data are consistent with what has been reported in the literature [13].

## 4. Materials and Methods

According to national regulations and institutional policies, ethical approval was not required due to the retrospective nature of the study, and it was conducted in compliance with the Declaration of Helsinki. The study was conducted on 54 FNAC samples of NHL and eight lymph node histological sections utilized as a control, collected at the Department of Pathology in San Giovanni di Dio e Ruggi d’Aragona Hospital with complete anonymity.

### 4.1. Fine-Needle Aspiration Cytology (FNAC)

Fine-needle aspiration cytology (FNAC) is routinely utilized in our Department for the triaging of lymphadenopathies. Criteria for patient enrollment, technical procedures, diagnoses, and cytological classifications were performed according to the Sydney System [30] and the WHO reporting system for lymph node cytopathology [31]. Namely, the diagnosis of NHL was achieved by combining FNAC features with flow cytometry (FC) on cell suspensions and/or immunocytochemistry (ICC) on formalin fixed cells on cell blocks. For FC, the basic panel included the following fluorochrome-conjugated antibodies: CD3, CD4, CD5, CD7, CD8, CD10, CD19, CD23, FMC7, and CD56, as previously described [32,33]. For ICC on cell blocks, a basic panel of 6–8 antibodies was selected based on specific cytological features, as previously described [34,35], from the following list: CD3, CD5, CD10, CD20, CD30, CD43, Bcl-1, Bcl-6, FOX1, Granzyme B, Perforin, IRTA, MUM1, SOX11, and Cyclin-D1. In 51 cases, specific NHL subtypes were identified by combining FNAC features and FC/ICC phenotypes. In three cases (cases no. 20, 21, and 48), FC or ICC either failed or were not performed due to scanty diagnostic material; consequently, the specific entities could not be identified, and a diagnosis of NHL-NOS was rendered. Since FNAC provided the initial diagnosis of NHL, all cases underwent histological confirmation. Histology confirmed the FNAC diagnoses, including the PTCL cases, and identified the specific histotype for case numbers 20, 21, and 48.

### 4.2. Samples

Inclusion criteria for case selection were as follows: FNAC diagnosis of NHL, confirmed diagnosis by histological control, availability of cytological material comprising at least three archived diagnostic slides, sample preparation performed between 2023 and 2025. A total of 54 cases fulfilled the inclusion criteria and were therefore selected for the study. In eight of these 54 cases, the corresponding formalin-fixed paraffin-embedded (FFPE) samples were also tested as control, so a total of 62 samples was included in the study. The 54 cytological samples consisted of stained slides obtained from FNAC of lymph nodes, including 24 cervical, 10 inguinal, 15 axillary and five supraclavicular. The study population included 54 patients: 19 males and 35 females, with a mean age of 65 years (range: 34 to 85 years). The cases were classified according to pathological diagnosis. Namely, there were diffuse large B-cell lymphoma (DLBCL) (29 cases), follicular lymphoma (FL) (14 cases), mantle cell lymphoma (MCL) (3 cases), Burkitt lymphoma (BL) (2 cases), peripheral T-cell lymphoma (PTCL) (2 cases), small lymphocytic lymphoma (SLL) (2 cases), and lymphoplasmacytic lymphoma (LPL) (2 cases). Samples and diagnoses are summarized in Table 2.

### 4.3. Extraction and Qualitative Analysis of RNA

Samples were subjected to RNA extraction using Magcore Plus II (RBC Bioscience, New Taipei City, Taiwan), a fully automated extractor based on magnetic beads for the specific binding of DNA/RNA while minimizing cross-contamination. The extraction took 3 h, and the final eluates were stored at −20 °C for sequencing up to seven days or at −80 °C for sequencing after more than a week. Extraction was executed as follows: using a scalpel blade 21, stained cytological slides and 4-micron white sections from FFPE were scraped to obtain small pellets. To each pellet, using sterile pipettes and tips exclusively for RNA, 500 µL of Sula Oil (a xylene-free buffer useful for deparaffinizing the samples), 20 µL of Proteinase K (necessary for digesting contaminating proteins), and 26 µL of DNase (necessary for digesting DNA) were added. These steps represent the only manual operations that must be performed by the operator. Afterward, the extractor needs to be closed until the RNA purification is complete. The extracted RNA obtained is ready for qualitative analysis. To ensure proper gene sequencing, we evaluated the quality of the individual samples to determine if they should be processed. For this purpose, the qualitative analysis of the target nucleic acid was performed using Myriapod RNA quantification strips, a kit from Diatech Pharmacogenetics (Jesi, Italy) that allows RNA quantification in terms of concentration (ng/µL) and an estimate of its integrity, which is useful and necessary to understand whether the nucleic acid is more or less fragmented. The use of fluorescence detection probes is the most accurate and reliable method, though costly, for the quantification of nucleic acids. The fluorescent probes used were FAM and HEX, probes of about 30 base pairs containing blue and green fluorescent dyes, respectively. The fluorescence signal increases significantly in direct proportion to the PCR product of the reaction. The amount of fluorescence acquired was processed as RNA concentration thanks to specific analysis software. The fragmentation index is the ratio of FAM and HEX concentrations, FAM binds to long fragments, HEX to short fragments, and FAM/HEX represents the fragmentation index of the examined nucleic acid. The result obtained was a value between 0 and 1: A fragmentation index between 0 and 0.3 indicates high fragmentation, suggesting that the sample has poor nucleic acid quality and is not intact, making it suboptimal for sequencing. High fragmentation is generally found in old samples or those that have undergone hyperfixation in the pre-analytical phase. In the case of high fragmentation but good nucleic acid concentration, the sample was considered suitable for subsequent sequencing because, despite being fragmented, it contained an adequate amount of RNA. A fragmentation index between 0.3 and 0.5 and between 0.5 and 1 indicated that the sample had medium and low nucleic acid fragmentation, respectively: for these samples, analytical errors are unlikely, except if low fragmentation is accompanied by a very low RNA concentration. According to the protocol, the minimum RNA input required to proceed with the subsequent phases (Fusion Plex Lymphoma—ArcherDX, Boulder, CO, USA) of analysis was 20 ng.

### 4.4. FusionPlex Lymphoma: Protocol and Procedures

**Figure 3:**
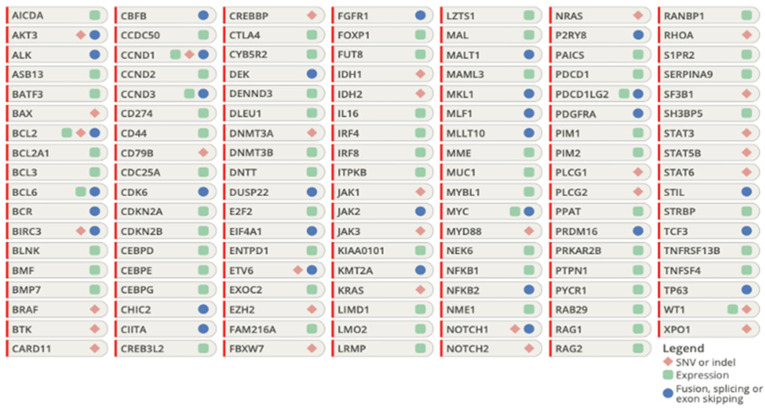
FusionPlex Lymphoma (ArcherDX, Boulder-USA) is a “Research Use Only” kit compatible with NGS—Illumina. This kit is designed to detect and identify gene fusions, point mutations and expression levels in 125 genes closely related to NHLs.

For gene sequencing, we utilized FusionPlex Lymphoma (ArcherDX, Boulder, CO, USA) a RUO kit compatible with the MiSeq-Illumina platform. This kit is designed to simultaneously detect and identify fusions, point mutations, and expression levels in 125 genes closely related to NHL. The 125 genes examined are shown in Figure 3. The principle of the method is enrichment based on Anchored Multiplex PCR (AMP). AMP is a form of targeted NGS that allows for the detection of fusions and the identification of partner genes from minimal nucleic acid inputs, approximately 10 ng per sample. After RNA extraction, complementary DNA (cDNA) is produced: cDNA is double-stranded DNA synthesized from a mature messenger RNA sample. In the AMP sequencing technique, the cDNA undergoes repair, adenylation, and ligation of the ends with a semi-functional universal adapter. The library is obtained after two successive PCR amplification cycles, which are the fundamental steps for target enrichment. The second phase of PCR uses primers for the universal sequencing adapter. The resulting target amplicons are functionalized for clonal amplification and subsequent sequencing.

### 4.5. Data Analysis

Data analysis was performed using the ArcherDX analysis platform (Archer Pipeline Versions 7.2.1-1; 7.3.0, 7.4.0, 7.4.1), an online bioinformatics tool that processes raw sequencing data and typically generates results within approximately 24 h. The operator uploaded the FASTQ files generated by MiSeq at the end of sequencing, along with the sample sheet, and then assigned a name to the analysis run for identification (e.g., Lymphomas1, Lymphomas2). For each sample, a report was generated with the following information: Sequencing compliance (green checkmark), which indicates whether the analysis was performed correctly; the presence or absence of genetic variants; details on the genes involved in the molecular alteration and allelic mutation frequency. The sequencing analysis software used facilitated the interpretation of NGS results even in the absence of bioinformaticians, simplifying the work. The following metrics were evaluated to assess sequencing quality for each sample: SS number (the number of unique star sites supporting the event ≧ 5), reads (the number of unique reads supporting the event ≧ 10), and % reads, which corresponds to the VAF (variant allele frequency) and is the percentage of reads supporting the event. Namely, the number of unique reads spanning the breakpoints and supporting the event divided by the total number of unique reads that span either breakpoint ≧ 10%, QC (threshold ≧ 50), total fragments passing quality filter (≧1.5 million), mapped fragments (≧1.5 million) and percent of fragments mapped (≧95%).

## 5. Conclusions

In conclusion, the NGS protocol for NHL used in this study is suitable for small samples in either cytological or FFPE. The technique is therefore flexible and ensures gene sequencing even for lower RNA inputs, providing valid results. The detected mutations were heterogeneous as anomalies categorized as “uncommon”, affecting less well-known genetic markers and not detectable with other methods, were found. The quality and quantity of data generated by NGS allow for better defining NOS and potentially provisional pathological entities [36]. Gene sequencing through NGS will enable a more accurate definition of individual entities [37], supporting and aiding in often complex diagnoses, and would serve as a foundation for new targeted therapies for NHL.

## Figures and Tables

**Table 1:** Diagnosis, RNA quality metrics, and molecular alterations detected in FNAC samples and corresponding histological controls.

N.	Diagnosis	RNA Concentration (FNAC)	Fragmentation Index (FNAC)	Fragmentation Quality (0–0.3 High/0.3–0.5 Medium/0.5–1.0 Low)	Mutations /Rearrangements (FNAC)	Mutations/Rearrangements (Histological Controls)	Mutations/Rearrangements (Literature Frequencies) (%)
1	DLBCL	0.03	1.00	LOW	not detected	not tested	n
2	DLBCL	36.2	0.37	MEDIUM	*LOC105370537-FUT8*	*LOC105370537-FUT8*	not described
3	FL	0.14	0.08	HIGH	not detected	not tested	
4	PTCL	17.0	0.34	MEDIUM	*EIF4E3-FOXP1*	*EIF4E3-FOXP1*	not described
5	DLBCL	3.10	0.01	HIGH	*EIF4A2-BCL6*	*EIF4A2-BCL6*	2%
6	FL	10.6	0.05	HIGH	*IGH-BCL6*	*IGH-BCL6*	10–15% [4]
7	DLBCL	1.20	0.29	HIGH	*MYD88* p.Val217Phe	*MYD88* p.Val217Phe	<1% [5]
8	SLL	6.00	0.20	HIGH	not detected	not tested	
9	DLBCL	41.0	0.54	LOW	not detected	not tested	
10	LPL	61.0	0.18	HIGH	not detected	not tested	
11	DLBCL	45.2	0.19	HIGH	not detected	not tested	
12	DLBCL	13.0	0.05	HIGH	not detected	not tested	
13	DLBCL	22.0	0.49	MEDIUM	not detected	not tested	
14	DLBCL	456	0.74	LOW	not detected	not tested	
15	FL	200	0.36	MEDIUM	not detected	not tested	
16	BL	23.0	0.35	MEDIUM	*TP53* p.Arg248Gln	*TP53* p.Arg248Gln	35% [6]
17	FL	10.0	0.20	HIGH	not detected	not tested	
18	MCL	4.50	0.32	MEDIUM	*IGH-CCND1*	*IGH-CCND1*	95% [7]
19	DLBCL	3.80	0.15	HIGH	not detected	not tested	
20	DLBCL	7.00	0.49	MEDIUM	not detected	not tested	
21	FL	0.03	1.00	LOW	not detected	not tested	
22	FL	36.0	0.39	MEDIUM	not detected	not tested	
23	PTCL	0.15	0.18	HIGH	*EIF4E3-FOXP1*	*EIF4E3-FOXP1*	not described
24	DLBCL	18.0	0.30	HIGH	*EIF4A2-BCL6*	*EIF4A2-BCL6*	<0.5% [8]
25	FL	8.00	0.20	HIGH	*IGH-BCL6*	*IGH-BCL6*	10–15% [4]
26	DLBCL	10.0	0.10	HIGH	not detected	not tested	
27	SLL	1.20	0.26	HIGH	not detected	not tested	
28	DLBCL	6.0	0.10	HIGH	not detected	not tested	
29	DLBCL	43.0	0.80	LOW	not detected	not tested	
30	LPL	40.0	0.50	LOW	not detected	not tested	
31	DLBCL	62.0	0.18	HIGH	not detected	not tested	
32	DLBCL	45.2	0.21	HIGH	not detected	not tested	
33	DLBCL	13.5	0.15	HIGH	not detected	not tested	
34	DLBCL	22.0	0.49	MEDIUM	not detected	not tested	
35	FL	156	0.74	LOW	not detected	not tested	
36	BL	200	0.37	MEDIUM	not detected	not tested	
37	DLBCL	24.0	0.45	MEDIUM	not detected	not tested	
38	MCL	10.0	0.21	HIGH	*IGH-CCND1*	*IGH-CCND1*	95% [7]
39	DLBCL	4.50	0.32	MEDIUM	not detected	not tested	
40	DLBCL	3.90	0.18	HIGH	*BCL2-IGH*	*BCL2-IGH*	15% [9]
41	MCL	7.30	0.48	MEDIUM	not detected	not tested	
42	DLBCL	0.07	0.25	HIGH	*IGH-BCL6*	*IGH-BCL6*	10–20% [10]
43	DLBCL	0.48	0.00	HIGH	not detected	not tested	
44	DLBCL	0.45	0.07	HIGH	*BCL2-IGH*	*BCL2-IGH*	15% [9]
45	DLBCL	6.07	0.00	HIGH	not detected	not tested	
46	DLBCL	11.3	0.12	HIGH	not detected	not tested	
47	FL	1.02	0.03	HIGH	*BCL2-IGH*	*BCL2-IGH*	90% [11]
48	DLBCL	2.03	0.08	HIGH	not detected	not tested	
49	FL	2.01	0.08	HIGH	*TBL1XR1-TP63*	*TBL1XR1-TP63*	1% [12]
50	FL	1.02	0.03	HIGH	not detected	not tested	
51	DLBCL	0.05	0.00	HIGH	*BCL2-IGH*	*BCL2-IGH*	15% [9]
52	FL	0.21	0.01	HIGH	not detected	not tested	
53	FL	0.07	0.00	HIGH	not detected	not tested	
54	FL	0.16	0.00	HIGH	not detected	not tested	

**Table 2:** Diagnostic distribution of non-Hodgkin lymphoma subtypes in cytological and histological samples.

Case N.	Lymph Node Site	FNAC Diagnosis	Immunofenotype (ICC, FC)	Histological Diagnosis
1	cervical	DLBCL	ICC	DLBCL
2	cervical	NHL nos	not performed	DLBCL
3	cervical	FL	FC	FL
4	cervical	PTCL	FC/ICC.	PTCL
5	inguinal	DLBCL	FC/ICC	DLBCL
6	inguinal	FL	FC	FL
7	inguinal	DLBCL	ICC	DLBCL
8	axillary	SLL	FC	SLL
9	axillary	DLBCL	FC/ICC	DLBCL
10	axillary	LPL	FC	LPL
11	inguinal	DLBCL	FC/ICC	DLBCL
12	cervical	DLBCL	ICC	DLBCL
13	supraclavicular	DLBCL	ICC	DLBCL
14	cervical	DLBCL	FC/ICC	DLBCL
15	cervical	FL	FC	FL
16	inguinal	BL	FC	BL
17	inguinal	FL	FC	FL
18	supraclavicular	MCL	FC/ICC	MCL
19	cervical	DLBCL	ICC	DLBCL
20	cervical	NHL nos	not performed	DLBCL
21	cervical	NHL nos	not performed	FL
22	cervical	FL	FC	FL
23	axillary	PTCL	FC/ICC	PTCL
24	axillary	DLBCL	ICC	DLBCL
25	cervical	FL	FC/ICC	FL
26	cervical	DLBCL	ICC	DLBCL
27	cervical	SLL	FC	SLL
28	cervical	DLBCL	ICC	DLBCL
29	cervical	DLBCL	FC/ICC	DLBCL
30	cervical	LPL	FC	LPL
31	inguinal	DLBCL	FC/ICC	DLBCL
32	axillary	DLBCL	FC/ICC	DLBCL
33	cervical	DLBCL	FC/ICC	DLBCL
34	cervical	DLBCL	ICC	DLBCL
35	cervical	FL	FC/ICC	FL
36	cervical	BL	FC/ICC	BL
37	supraclavicular	DLBCL	ICC	DLBCL
38	cervical	MCL	FC/ICC	MCL
39	supraclavicular	DLBCL	ICC	DLBCL
40	inguinal	DLBCL	FC/ICC	DLBCL
41	inguinal	MCL	FC/ICC	MCL
42	inguinal	DLBCL	FC/ICC	DLBCL
43	cervical	DLBCL	ICC	DLBCL
44	axillary	DLBCL	FC/ICC	DLBCL
45	axillary	DLBCL	FC/ICC	DLBCL
46	axillary	DLBCL	FC/ICC	DLBCL
47	axillary	FL	FC/ICC	FL
48	axillary	NHL nos	not performed	DLBCL
49	supraclavicular	FL	FC/ICC	FL
50	axillary	FL	FC	FL
51	axillary	DLBCL	FC/ICC	DLBCL
52	axillary	FL	FC/ICC	FL
53	axillary	FL	FC/ICC	FL
54	cervical	FL	FC	FL

## Data Availability

The original contributions presented in this study are included in the article. Further inquiries can be directed to the corresponding authors.
